# Exploring the Link between Serum Phosphate Levels and Low Muscle Strength, Dynapenia, and Sarcopenia

**DOI:** 10.1038/s41598-018-21784-1

**Published:** 2018-02-23

**Authors:** Yuan-Yuei Chen, Tung-Wei Kao, Cheng-Wai Chou, Chen-Jung Wu, Hui-Fang Yang, Ching-Huang Lai, Li-Wei Wu, Wei-Liang Chen

**Affiliations:** 1 Department of Internal Medicine, Tri-Service General Hospital Songshan Branch, School of Medicine, National Defense Medical Center, Taipei Taiwan, Republic of China; 2 Division of Family Medicine, Department of Family and Community Medicine, Tri-Service General Hospital and School of Medicine, National Defense Medical Center, Taipei Taiwan, Republic of China; 3 Division of Family Medicine, Department of Community Medicine, Taoyuan Armed Forces General Hospital, Taoyuan Taiwan, Republic of China; 4 Division of Geriatric Medicine, Department of Family and Community Medicine, Tri-Service General Hospital and School of Medicine, National Defense Medical Center, Taipei Taiwan, Republic of China; 5 Graduate Institute of Clinical Medical, College of Medicine, National Taiwan University, Taipei Taiwan, Republic of China; 6 School of Public Health, National Defense Medical Center, Taipei, Republic of China

## Abstract

Emerging evidences addressed an association between phosphate and muscle function. Because little attention was focused on this issue, the objective of our study was to explore the relationship of phosphate with muscle strength, dynapenia, and sarcopenia. From the National Health and Nutrition Examination Survey, a total of 7421 participants aged 20 years or older were included in our study with comprehensive examinations included anthropometric parameters, strength of the quadriceps muscle, and appendicular lean masses. Within the normal range of serum phosphate, we used quartile-based analyses to determine the potential relationships of serum phosphate with dynapenia, and sarcopenia through multivariate regression models. After adjusting for the pertinent variables, an inverse association between the serum phosphate quartiles and muscle strength was observed and the linear association was stronger than other anthropometric parameters. Notably, the significant association between phosphate and muscle strength was existed in >65 years old age group, not in 20–65 years old. The higher quartiles of phosphate had higher likelihood for predicting the presence of dynapenia rather than sarcopenia in entire population. Our study highlighted that higher quartiles of phosphate had significant association with lower muscle strength and higher risks for predicting the presence of dynapenia.

## Introduction

Aging is a complicated process that results in muscle weakness and disability due to variations in skeletal muscle quantity and quality^1^. The terms of “sarcopenia” is limited to its original definition of an age-related loss of skeletal muscle mass, and “dynapenia” is defined as the present the age-related loss of strength^2^. It has been proposed that muscle strength is a more useful index of the development of declines in mobility and disability than muscle mass^3^. Stenholm *et al*. demonstrated that low muscle strength could predict the risk of death in adult men and women with an additive pattern^4^. A threshold of muscle strength associated with an increased likelihood of MetS in men was identified in a large-scale study^5^. Muscle strength is included in the definition of frailty status, which is closely associated with HRQoL in elderly Taiwanese preventive health service users^6^.

Serum phosphate is absorbed through the gut, stored in the bone and reabsorbed through the proximal renal tubules^7^. The level of serum phosphate is regulated within a narrow range in healthy individuals because inorganic phosphate plays fundamental physiological roles in energy production, membrane transport, and signal transduction^8^. Inorganic phosphate remains relatively constant through the influence of multiple factors, such as parathyroid hormone, fibroblast growth factor 23, and vitamin D, on the kidneys, bone, and digestive system^9^. In patients with chronic kidney disease (CKD), the kidneys fail to excrete excessive phosphate, which leads to a hyperphosphatemic state^10^.

In a recent literature, Acevedo *et al*. demonstrated that high-phosphorus diet potentiated adverse skeletal muscle changes in long-term uremic rats^11^. Inorganic phosphate had been observed to play an important role in muscle contractile function^12^, and increased inorganic phosphate was involved in skeletal muscle fatigue^13^. The objects of our study were to examine the association between phosphate with muscle strength and whether the phosphate could predict the presence of dynapenia or sarcopenia based on population-based data analysis from the NHANES 1999–2002.

## Results

### Serum phosphate level and different anthropometric parameters

The associations of the serum phosphate level and different anthropometric parameters (MS, TC, CC, and ALM) were analyzed as shown in Table 1. Notably, the serum phosphate level in the normal range had significant negative relationships with MS, TC and CC (*P* < 0.05), but not in the hyperphosphatemic or hypophosphatemic individuals. The fully adjusted β coefficient for the serum phosphate with the MS, TC and CC were −28.37, −1.99 and −1.12 (95% confidence intervals (95%CI) = −52.23, −4.50, *P* < 0.05; 95%CI = −3.05, −0.93, P < 0.001; and 95%CI = −1.78, −0.47, *P* < 0.001, respectively). In other words, the subjects with higher serum phosphate tended to have lower MS, TC and CC in our findings.

**Table 1:** Association between the serum phosphate and the anthropometric parameters.

Anthropo-metric Parameters	Model^a^ 1 β^b^ (95% CI)	*P* Value	Model^a^ 2 β^b^ (95% CI)	*P* Value	Model^a^ 3 β^b^ (95% CI)	*P* Value
Normal range of serum phosphates						
Muscle Strength (nt)	−80.89 (−112.85, −48.93)	<0.001	−28.05 (−51.89, −4.20)	0.021	−28.37 (−52.23, −4.50)	0.020
Appendicular lean mass (g)	−2758 (−3724, −1791)	<0.001	−578 (−1257, 99)	0.095	−544 (−1220, 132)	0.115
TC (cm)	−0.62 (−1.71, 0.48)	0.270	−2.04 (−3.10, −0.98)	<0.001	−1.99 (−3.05, −0.93)	<0.001
CC (cm)	−1.08 (−1.74, −0.42)	<0.001	−1.17 (−1.83, −0.51)	<0.001	−1.12 (−1.78, −0.47)	<0.001
Serum phosphates <0.81						
Muscle Strength (nt)	−426.99 (−991.66, 137.68)	0.136	−191.68 (−624.36, 241.00)	0.379	108.36 (−466.40, 683.12)	0.707
Appendicular lean mass (g)	−16837 (−33741, 66)	0.051	−1158 (−13476, 11159)	0.853	−5628 (−18396, 7139)	0.385
TC (cm)	−16.47 (−38.16, 5.22)	0.136	−14.14 (−35.39, 7.12)	0.191	−15.39 (−37.31, 6.53)	0.167
CC (cm)	−5.62 (−18.62, 7.38)	0.395	−5.52 (−18.58, 7.54)	0.405	−7.85 (−21.37, 5.68)	0.253
Serum phosphate >1.49						
Muscle Strength (nt)	−174.38 (−445.92, 97.15)	0.202	−160.93 (−410.89, 89.03)	0.200	−193.23 (−437.47, 51.00)	0.116
Appendicular lean mass (g)	−1578 (−7743, 4587)	0.614	1854 (−3831, 7541)	0.521	2274 (−3642, 8192)	0.449
TC (cm)	−6.47 (−12.64, −0.30)	0.040	−1.84 (−9.91, 6.22)	0.653	−0.91 (−9.13, 7.32)	0.828
CC (cm)	−3.96 (−7.98, 0.07)	0.054	−0.19 (−5.45, 5.07)	0.943	0.25 (−5.17, 5.67)	0.928

^a^Adjusted covariates:

Model 1 = Unadjusted.

Model 2 = Model 1 + age, sex, race/ethnicity, BMI, systolic blood pressure, serum fasting glucose, serum cholesterol, serum albumin, ALT, serum uric acid, C-reactive protein.

Model 3 = Model 2 + history of congestive heart failure, coronary heart disease, angina/angina pectoris, heart attack, stroke, cancer/malignancy, smoking, moderate to vigorous recreational activity.

^b^β coefficients was interpreted as change of telomere length for each increase in different anthropometric parameters.

Abbreviation:

TC, thigh circumference; CC, calf circumference.

### Demographic characteristics of the eligible subjects

In Table 2, participants with frankly low (<0.81) and high (>1.49) phosphate levels and normal range divided into quartiles were categorized and the characteristics of these subgroups were subsequently summarized. The serum phosphate level was significantly associated with age, BMI, total cholesterol, serum high density lipoprotein, serum fasting glucose, serum calcium and serum creatinine. In normal range of serum phosphate, the muscle strength of participants in the higher quartiles were weaker than those in the lower quartiles. Besides, subjects with hyperphosphatemia had lower muscle strength than those with normal range and with hypophosphatemia.

**Table 2:** Characteristics of study participants of quartiles of serum phosphate.

Characteristics of Study Participants	Hypophosphatemia	Quartiles of serum phosphate in normal range				Hyperphosphatemia	*P* Value
	<0.81	Q1 (0.81–1.03) (n = 1880)	Q2 (1.03–1.16) (n = 2018)	Q3 (1.16–1.26) (n = 1869)	Q4 (1.29–1.49) (n = 1654)	>1.49	
**Continuous variables, mean (SD)**							
Age (years)	53.24(16.44)	52.29(18.16)	50.51(18.79)	48.65(19.01)	49.62(18.86)	41.19(18.82)	<0.001
Thigh circumference (cm)	53.37(7.43)	52.99(7.06)	53.12(7.21)	52.66(7.01)	52.73(7.07)	53.33(7.03)	0.264
Calf circumference (cm)	38.32(4.59)	38.33(4.30)	38.36(4.31)	37.95(4.25)	37.96(4.38)	38.35(4.59)	0.010
Muscle Strength (nt)	401.04(126.00)	365.76(120.09)	350.09(124.92)	338.14(115.83)	331.78(124.69)	303.36(122.24)	<0.001
Appendicular lean mass (g)	23066.66(5910.36)	22424.09(5858.85)	21988.30(6168.79)	21335.16(6082.25)	21412.85(6422.66)	22969.98(6802.24)	<0.001
BMI (kg/m^2^)	29.20(6.59)	28.79(6.11)	28.36(6.20)	28.07(6.04)	27.98(6.21)	28.44(6.17)	<0.001
AST (U/L)	28.22(30.94)	25.04(16.51)	24.24(12.81)	24.71(24.12)	24.64(15.72)	35.15(123.19)	0.563
Serum TC (mg/dL)	192.60(41.66)	199.94(40.85)	198.54(38.64)	201.86(42.94)	202.30(41.54)	206.95(48.28)	0.017
Serum FG (mg/dL)	107.26(36.09)	101.77(39.53)	96.28(30.52)	96.18(35.88)	94.90(34.84)	91.10(29.89)	<0.001
Serum HDL (mg/dL)	46.93(13.22)	50.05(15.17)	51.60(15.89)	52.54(15.56)	52.75(15.91)	52.47(16.40)	<0.001
Calcium (mg/dL)	9.26(0.53)	9.32(0.43)	9.39(0.39)	9.43(0.39)	9.46(0.40)	9.56(0.49)	<0.001
Creatinine (mg/dL)	0.82(0.33)	0.81(0.27)	0.84(0.39)	0.80(0.42)	0.85(0.47)	1.25(1.69)	<0.001
**Categorical variables, n (%)**							
Arthritis	48(2.5)	490(27.1)	509(28.2)	439(24.3)	369(20.4)	46(2.4)	0.050
Congestive heart failure	8(3.5)	64(30.8)	54(26)	56(26.9)	34(16.3)	14(6.1)	0.204
Coronary heart disease	8(2.4)	98(32.2)	83(27.3)	73(24)	50(16.4)	17(5.2)	0.129
Angina/angina pectoris	11(4.0)	74(28.9)	76(29.7)	67(26.2)	39(15.2)	14(5.0)	0.083
Heart attack	10(3.0)	90(28.8)	83(26.6)	86(27.6)	53(17)	14(4.2)	0.127
Stroke	8(3.4)	64(28.2)	59(26)	49(21.6)	55(24.2)	3(1.3)	0.697
Smoking	84(2.2)	924(25.7)	973(27.1)	865(24.1)	828(23.1)	133(3.5)	0.352

BMI, body mass index; SBP, systolic blood pressure; Serum FG, serum fasting glucose; Serum TC, serum total cholesterol; AST, aspartate aminotransferase; Serum HDL, serum high density lipoprotein.

^a^Values were expressed as mean (standard deviation).

^b^Values in the categorical variables were expressed as number (%).

### Association between phosphate and muscle strength

The quartile-based analysis of not only normal range of serum phosphate but also abnormal range was conducted by multiple linear regression, and the results are presented in Table 3. In normal range, negative associations between phosphate with muscle strength and TC were noted among all models. The higher quartiles of serum phosphate had lower muscle strength and TC, and a dose-dependent response was noted. However, no significant differences were found between serum phosphate with all anthropometric parameters in the hyperphosphatemic or hypophosphatemic groups.

**Table 3:** Association between the Muscle strength and serum phosphate.

Variables		Models								
		Model 1			Model 2			Model 3		
Anthropometric parameters	Quartiles	β^b^ (95% CI)	*P* Value	*P* for Trend	β^b^ (95% CI)	*P* Value	*P* for Trend	β^b^ (95% CI)	*P* Value	*P* for Trend
Normal range of serum phosphate										
Thigh Circumference	Q2 v.s. Q1	−0.26(−0.69, 0.18)	0.247	<0.001	0.07(−0.13, 0.27)	0.491	<0.001	0.07(−0.13, 0.28)	0.472	<0.001
	Q3 v.s. Q1	−0.81(−1.26, −0.37)	<0.001		−0.14(−0.35, 0.06)	0.173		−0.14(−0.35, 0.07)	0.182	
	Q4 v.s. Q1	−1.00(−1.46, −0.54)	<0.001		−0.32(−0.54, −0.10)	0.004		−0.31(−0.52, −0.09)	0.006	
Calf Circumference	Q2 v.s. Q1	−0.09(−0.36, 0.18)	0.504	<0.001	−0.15(−0.01, 0.30)	0.054	0.228	0.154(0.01, 0.31)	0.047	0.278
	Q3 v.s. Q1	−0.47(−0.74, −0.19)	<0.001		−0.03(−0.19, 0.13)	0.703		−0.03(−0.18, 0.13)	0.730	
	Q4 v.s. Q1	−0.55(−0.83, −0.26)	<0.001		−0.04(−0.21, 0.12)	0.602		−0.03(−0.20, 0.13)	0.699	
Muscle Strength	Q2 v.s. Q1	−7.66(−16.89, 1.57)	0.104	0.033	−7.08(−16.08, 1.92)	0.123	0.034	−6.48(−15.48, 2.52)	0.158	0.035
	Q3 v.s. Q1	−9.62(−19.20, −0.04)	0.049		−8.90(−18.28, 0.47)	0.063		−8.47(−17.84, 0.89)	0.076	
	Q4 v.s. Q1	−10.47(−20.92, −0.01)	0.050		−10.44(−20.71, −0.16)	0.047		−10.39(−20.66, −0.12)	0.047	
Serum phosphate <0.81										
Thigh Circumference	Q2 v.s. Q1	−0.98 (−4.00, 2.05)	0.525	0.626	−1.68 (−4.62, 1.26)	0.261	0.832	−1.29 (−4.20, 1.61)	0.380	0.932
	Q3 v.s. Q1	−2.47 (−5.42, 0.49)	0.101		−1.95 (−4.87, 0.98)	0.191		−1.47 (−4.31, 1.38)	0.310	
	Q4 v.s. Q1	−0.42 (−3.06, 3.91)	0.811		0.54 (−2.87, 3.94)	0.756		0.58(−2.77, 3.93)	0.732	
Calf Circumference	Q2 v.s. Q1	−1.01 (−2.86, 0.85)	0.284	0.635	−1.49 (−3.31, 0.33)	0.108	0.853	−1.28 (−3.09, 0.53)	0.164	0.746
	Q3 v.s. Q1	−0.96 (−2.76, 0.84)	0.295		−0.82 (−2.63, 0.98)	0.369		−0.75 (−2.52, 1.01)	0.400	
	Q4 v.s. Q1	−0.27 (−2.34, 1.81)	0.802		−0.08 (−2.12, 1.96)	0.936		−0.24 (−2.25, 1.77)	0.815	
Muscle Strength	Q2 v.s. Q1	−5.53 (−87.62, 76.56)	0.893	0.105	−18.84 (−84.28, 46.61)	0.566	0.811	7.16 (−65.29, 79.60)	0.843	0.766
	Q3 v.s. Q1	−25.57(−109.07, 57.92)	0.543		1.38 (−76.41, 79.17)	0.972		38.16(−50.29, 126.61)	0.390	
	Q4 v.s. Q1	−73.22(−162.02, 15.57)	0.104		−12.25(−81.21, 56.70)	0.723		7.61(−66.99, 82.21)	0.838	
Serum phosphate >1.49										
Thigh Circumference	Q2 v.s. Q1	1.68 (−0.97, 4.34)	0.213	0.462	2.16 (−0.33, 4.65)	0.088	0.841	2.89 (0.35, 5.44)	0.026	0.940
	Q3 v.s. Q1	0.63 (−2.14, 3.41)	0.653		1.34 (−1.22, 3.91)	0.303		1.73 (−0.90, 4.37)	0.196	
	Q4 v.s. Q1	−0.36 (−3.14, 2.43)	0.802		0.32 (−2.36, 2.99)	0.817		0.77 (−1.97, 3.50)	0.582	
Calf Circumference	Q2 v.s. Q1	1.19 (−0.52, 2.90)	0.170	0.479	1.29 (−0.36, 2.95)	0.125	0.916	1.67 (−0.03, 3.37)	0.054	0.842
	Q3 v.s. Q1	0.45 (−1.34, 2.23)	0.623		0.89 (−0.82, 2.59)	0.306		1.08 (−0.67, 2.84)	0.226	
	Q4 v.s. Q1	−0.19 (−1.98, 1.60)	0.839		0.21 (−1.56, 1.98)	0.817		0.26 (−1.56, 2.07)	0.782	
Muscle Strength	Q2 v.s. Q1	30.27(−70.87, 131.41)	0.549	0.274	1.52 (−72.89, 75.94)	0.967	0.125	3.63 (−73.32, 80.58)	0.923	0.054
	Q3 v.s. Q1	−60.00(−175.66, 55.66)	0.301		−73.21(−157.81, 11.39)	0.087		−58.70(−144.91, 27.50)	0.173	
	Q4 v.s. Q1	−27.44(−143.10, 88.21)	0.634		−40.28(−121.61, 41.05)	0.321		−58.80(−142.86, 25.26)	0.162	

^a^Adjusted covariates:

Model 1 = Unadjusted.

Model 2 = Model 1 + age, sex, race/ethnicity + BMI, serum fasting glucose, serum cholesterol, AST, serum HDL, serum calcium, serum creatinine.

Model 3 = Model 2+ history of congestive heart failure, coronary heart disease, angina/angina pectoris, heart attack, stroke, smoking.

^b^β coefficients was interpreted as change of telomere length for each increase in different anthropometric parameters.

Abbreviation: BMI, body mass index; SBP, systolic blood pressure; ALT, alanine aminotransferase.

In Table 4, we further examine the association of muscle strength and serum phosphate level according to age. A significant relationship was observed in participants older than 65 years (P value, 0.011, 0.021 and 0.021 respectively).

**Table 4:** Association between the serum phosphate and the muscle strength in elderly participants.

Anthropo-metric Parameters	Model^a^ 1 β^b^ (95% CI)	*P* Value	Model^a^ 2 β^b^ (95% CI)	*P* Value	Model^a^ 3 β^b^ (95% CI)	*P* Value
<65 y/o						
Muscle Strength	−15.68 (−49.64, 18.28)	0.365	−23.48 (−57.24, 10.29)	0.173	−21.52 (−55.11, 12.06)	0.209
≥65 y/o						
Muscle Strength	−39.43 (−69.79, −9.06)	0.011	−34.92 (−64.68, −5.16)	0.021	−35.17 (−65.01, −5.33)	0.021

Model 1 = Unadjusted.

Model 2 = Model 1 + age, sex, race/ethnicity + BMI, serum fasting glucose, serum cholesterol, AST, serum HDL, serum calcium, serum creatinine.

Model 3 = Model 2 + history of congestive heart failure, coronary heart disease, angina/angina pectoris, heart attack, stroke, smoking.

The odd ratios for dynapenia and sarcopenia performed by the multivariable-adjusted logistic regression with phosphate quartiles analysis were provided in Table 5. In dynapenia, the higher quartiles of phosphate had higher risks for predicting the presence of dynapenia for all models in both 20–65 and >65 years old age groups. In sarcopenia, however, the same trend was only found in the unadjusted model for both age groups. The significant differences were existed in the highest quartile of phosphate in adjusted models with sarcopenia in 20–65 years old age group, but not in >65 years old.

**Table 5:** Association between the quartiles of serum phosphate and the presence of dynapenia and sarcopenia.

Variables	Models						
	Model 1			Model 2			Model 3
Age group	Quartiles	OR (95% CI)	*P* Value	OR (95% CI)	*P* Value	OR (95% CI)	*P* Value
**Dynapenia**							
20–65 years old	Q2 v.s. Q1	1.37(0.99–1.89)	0.06	1.42(1.02–1.99)	0.04	1.37(0.98–1.93)	0.07
	Q3 v.s. Q1	1.69(1.24–2.31)	<0.001	1.74(1.26–2.40)	<0.001	1.67(1.20–2.32)	0.003
	Q4 v.s. Q1	1.60(1.15–2.24)	0.006	1.82(1.28–2.60)	<0.001	1.80(1.25–2.58)	<0.001
>65 years old	Q2 v.s. Q1	1.62(1.16–2.27)	0.005	1.64(1.16–2.33)	0.006	1.57(1.10–2.23)	0.013
	Q3 v.s. Q1	1.86(1.31–2.62)	<0.001	1.76(1.23–2.51)	0.002	1.71(1.19–2.46)	0.004
	Q4 v.s. Q1	2.25(1.55–3.27)	<0.001	2.24(1.52–3.31)	<0.001	2.13(1.43–3.16)	<0.001
**Sarcopenia**							
20–65 years old	Q2 v.s. Q1	1.13(0.96–1.33)	0.151	1.10(0.88–1.38)	0.400	1.11(0.88–1.33)	0.376
	Q3 v.s. Q1	1.37(1.17–1.61)	<0.001	1.13(0.90–1.41)	0.285	1.13(0.90–1.41)	0.297
	Q4 v.s. Q1	1.40(1.19–1.65)	<0.001	1.27(1.01–1.61)	0.043	1.28(1.01–1.62)	0.038
>65 years old	Q2 v.s. Q1	1.53(1.16–2.02)	0.003	1.11(0.75–1.65)	0.595	1.14(0.77–1.70)	0.513
	Q3 v.s. Q1	1.99(1.50–2.64)	<0.001	1.20(0.81–1.78)	0.371	1.24(0.83–1.85)	0.297
	Q4 v.s. Q1	2.63(1.95–3.55)	<0.001	1.45(0.95–2.20)	0.085	1.47(0.96–2.25)	0.074

Model 1 = Unadjusted.

Model 2 = Model 1 + age, sex, race/ethnicity + BMI, serum fasting glucose, serum cholesterol, AST, serum HDL, serum calcium, serum creatinine.

Model 3 = Model 2 + history of congestive heart failure, coronary heart disease, angina/angina pectoris, heart attack, stroke, smoking.

### Association between entire range of serum phosphate and muscle strength

In Supplementary Tables 1–5, we investigated the association of entire range of phosphate levels and muscle strength without excluding frankly low and high phosphate. After additionally adjusting for other covariates from Models 1 to 4, the inverse association between muscle strength and serum phosphate remained essentially unchanged. The similar results were consistent with the original findings in our study.

### Gender difference in association between phosphate and muscle strength

In Supplementary Tables 6–11, gender difference was performed to examine the association of phosphate with muscle strength and dynapenia instead of age variable. In male, muscle strength had negative association with 3rd quartile of phosphate in Model 2 and Model 3 (Supplementary Table 10**)**. In female, muscle strength was associated with 2nd and 4th quartiles of phosphate in Model 2 and Model 3. To predict the presence of dynapenia and sarcopenia, logistic regression was performed with phosphate divided into quartiles. In male, the higher quartiles of phosphate were significantly associated with the presence of dynapenia after model adjustment (Supplementary Table 11). In female, 4^th^ quartile of phosphate had association with dynapenia in Model 2 and Model 3. However, the presence of sarcopenia was only predicted by 4^th^ quartile of phosphate with model adjustment in female group.

## Discussion

Based on this population-based, non-institutionalized sample of U.S. citizens, our study demonstrated an inverse association between serum phosphate and muscle strength. Furthermore, the higher levels of phosphate had higher risks to predict the presence of dynapenia in both age groups. To the best of our knowledge, this study is the first to provide epidemiological evidence regarding the relationship of serum phosphate and dynapenia.

Hypophosphatemia resulted in skeletal muscle damage with an elevation of the serum creatine phosphokinase and rhabdomyolysis^14^. Besides, alternation in renal acid–base status caused by hypophosphatemia via depressed proximal tubular reabsorption of bicarbonate and decreased buffer excretion due to low phosphate excretion^15^. Hypophosphatemia could contribute to muscle weakness and respiratory and heart failure^16^. In a study of mice model, muscle weakness could be explained by decreased muscle ATP synthetic flux which caused by hypophosphatemia^17^. Hyperphosphatemia could result from increased phosphate intake, decreased phosphate excretion, or a disorder that shifts intracellular phosphate to extracellular space. In the hyperphosphatemic mouse models, force decline of intact muscle was accompanied by increased inorganic phosphate and reduced tetanic myoplasmic Ca^2+^ ^18^.

For hyperphosphatemia, the possible underlying physiological functions of phosphate on muscle function could be divided into direct and indirect pathways. Emerging evidences had indicated that hyperphosphatemia led to vascular calcification with mineral deposition in vessel wall, leading to muscle dysfunction. It also caused related CVD mortalities including coronary artery disease and peripheral arterial disease (PAD) in CKD patients^19,20^. Patients with PAD manifested altered muscle control strategies and further strength deficits^21^. McDermott *et al*. represented that weaker plantar flexion strength, knee extension power, and hand grip strength were correlated with increased mortality in participants with PAD^22^. Phosphate was directly and independently correlated with inflammation^23^. Clinical studies had reported that serum phosphate was associated with serum inflammatory markers in CKD patients^24^ and phosphate overload directly induced systemic inflammatory and malnutrition in CKD rats^25^. High IL-6 and CRP levels were resulted in threefold greater risk of losing 40% of grip strength^26^. It was similar with our findings that elevated serum phosphate had significant associated with decline of muscle strength in participants with elevated CRP levels (Supplementary Table 14). It raised the possibility that that inflammatory processes mediated the association between serum phosphate and muscle strength. The possible mechanism was that inflammatory markers could induce alterations of muscle proteins independently of protein loss and result in diminished force production^27^. Several indirect consequences resulted in disturbance of phosphate homeostasis and caused hyperphosphatemia. Klotho-fibroblast growth factor system was thought to play an important role in the control of serum phosphate^28^. Hyperphosphatemia indirectly increased parathyroid hormone (PTH) secretion to reduce serum calcium by different possible pathways. First, increased serum phosphate contributed to calcium precipitation in soft tissues. Second, it might increase resistance of bone to PTH action by decreasing the efflux of calcium from bone. Third, it also impeded the 1 a-hydroxylase enzyme to reduce the levels of 1, 25(OH)2D3 (calcitriol)^29^. Low calcitriol levels resulted in decreased intestinal absorption of calcium and hypocalcemia^30^. Failure of sarcoplasmic reticulum Ca^2+^ release was proposed to be a major contributor to muscle fatigue^12^. In a recent study, hypocalcemia was reported to be one of the factors causing the decline in leg muscle strength in athletes^31^.

In terms of hypophosphatemia, the effect of hypophosphatemia on muscle function was examined in dog that reversible changes in the skeletal muscle composition and transmembrane potential was caused by moderate phosphorus depletion^32^. It was well known that a deficiency of vitamin D resulted in muscle weakness and a significant reduction in muscle force was noted when vitamin D deficiency was accompanied by hypophosphatemia^33^. Klotho and fibroblast growth factor were proposed that might play an important role in the maintenance of muscle property and function by interplaying vitamin D and phosphate in energy (ATP) and protein production^34^. Another association between muscle and hypophosphatemia was hypophosphatemic osteomalacia. Proximal muscle weakness and low serum phosphorus concentrations were common characteristic of these patients^35^.

In our study, higher levels of phosphate were significantly associated with the decline of muscle strength, but not the decrease of lean muscle mass. Higher quartiles of phosphate had higher risks of predicting the presence of dynapenia rather than sarcopenia. It seemed that phosphate might influence the decline of muscle strength via some potential pathways such as changes in neuromuscular transmission, muscle architecture, and the processes involved in E-C coupling instead of the change of muscle mass. It was generally accepted that reduction of muscle force occurred because phosphate was released from cross-bridges at a stage closely associated with force production so that elevation of phosphate accelerated the backward rate of this step and thus reduced force^36,37^. Neuromuscular fatigue onset was associated with an alteration of the mechanisms involved in force production which might be induced by a perturbation of the calcium movements, and accumulation of phosphate^38^. It was already proposed that increasing inorganic phosphate reduced cross-bridge force and Ca^2+^ sensitivity of the myofilaments^39^. Increasing the open probability of the SR Ca^2+^ release channels and slowing of the SR Ca^2+^ pump might be plausible causes of muscle fatigue^40,41^. It had long been known that cytoplasmic inorganic phosphate could cross into the SR and precipitate with the Ca^2+^ there. Precipitation of Ca^2+^ and phosphate in the SR would not only reduce the amount of Ca^2+^ available for release, but would also decrease the stimulatory effect of SR Ca^2+^ on the release channel^42^. However, no mechanistic explanation could be provided based on the available data.

There are still several limitations concerning the current study. First, the interpretations cannot be extended to causal inferences because this study was a cross-sectional observational analysis of an existing database. The long-term association between muscle strength decline and fluctuations in serum phosphate level was not investigated because repeated observations of these clinical variables were not recorded. Second, paucity of unmeasured variables such as parathyroid hormone and fibroblast growth factor-23 in NHANES 1999–2002 dataset might cause incomprehensive analysis. Because the data of serum vitamin D was only available in the NHANES 2001–2002, we tried to reanalysis our modeling hypothesis. After additional adjustment of serum vitamin D, the inverse association between serum phosphate and muscle Strength remained unchanged (Supplemental Tables 12 and 13). Third, data related to potentially confounding biomarkers such as IL-6 and TNF-alpha are not accessible in the NHANES database. If we could analyze these inflammatory biomarkers, which influence muscle strength as reported in previous studies, interesting findings could be uncovered. Finally, the complete pathway and mechanisms of increments in serum phosphate levels were not fully addressed.

## Conclusion

Our study highlighted that being within the higher quartiles of phosphate levels within the normal range was inversely associated with muscle strength and had higher risks for predicting the presence of dynapenia. Hyperphosphatemia may result in impaired functions of the muscles that harbor a predisposing milieu for the decline of muscle strength and development of dynapenia. Although no prospective randomized controlled trial was assessable, our study provides epidemiologic evidence and a plausible pathophysiological mechanism for future studies to use to determine a strategy for the prevention of dynapenia and physical disabilities.

## Methods

### Ethics statement

The NHANES study protocol was conducted according to the National Center for Health Statistics (NCHS) Institutional Review Board (IRB). Before data collection and the health examinations, all informed consents had been obtained. All the experimental protocols were approved by NCHS IRB.

### Study design and participants

All data were obtained from the National Health and Nutrition Examination Survey (NHANES), which was a cross-sectional survey of a representative sample of the U.S. population from 1999 to 2002 that was administered by the National Center for Health Statistics (NCHS) of the Centers for Disease Control and Prevention (CDC). The NHANES collected representative data on the health and nutritional statuses of non-institutionalized civilian residents and conducted more detailed laboratory analyses on a subset of the participants. These data are available for public download.

**Figure 1:**
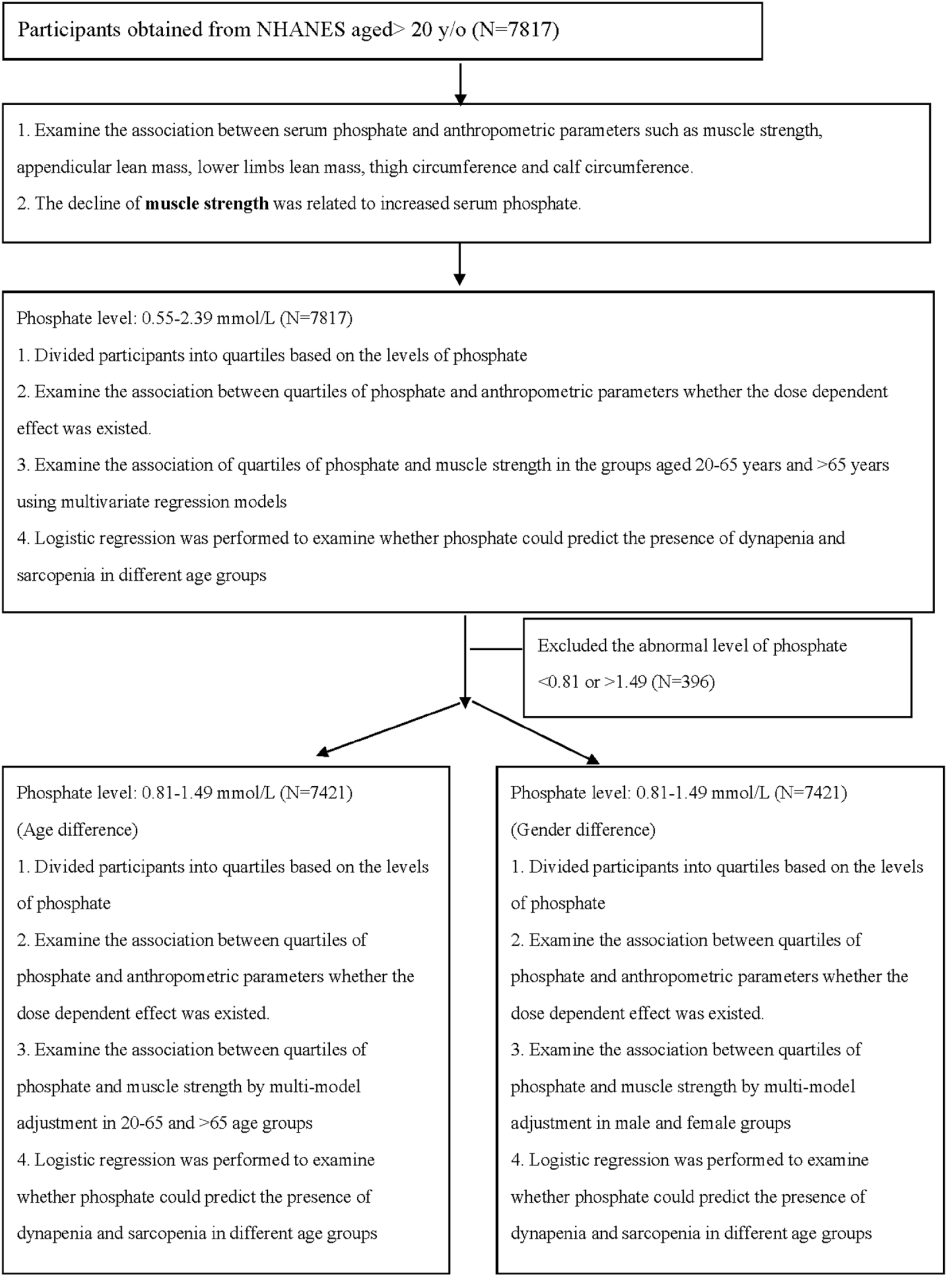
Study flow diagram.

According to the flow chart of the study (Fig. 1), our study sample was composed of 7817 participants who were 20 years old and older. We excluded those with serum phosphate levels <0.81 mg/dL or >1.49 mg/dL to reduce any confounding effects and finally 7421 participants were analyzed in further stages. After examining the association between phosphate and anthropometric parameters, the decline of muscle strength was a better index which related to increased phosphate compared to other parameters. Normal phosphate levels were divided into quartiles to examine the association with muscle strength, thigh and calf circumference by multivariable model adjustment and observe whether the dose dependent effects were existed. Next, we examined the association of phosphate and muscle strength in 20–65 years old and >65 years old age groups respectively. Logistic regression was performed to analyze the odd ratios whether quartiles of phosphate predicted the presence of dynapenia and sarcopenia in different age groups in the final stage.

### Measurement of serum phosphate

Serum phosphate was measured with a Hitachi model 737 multichannel analyzer (Boehringer Mannheim Diagnostics, Indianapolis, IN). Inorganic phosphate was reacted with ammonium molybdate in an acidic solution to construct ammonium phosphomolybdate, which was quantified in the ultraviolet range (340 nm) using a sample-blanked end point method^43^.

### Assessment of anthropometric parameters

The calf and thigh circumference were measured by using an elastic tape and an elastic measuring tape was placed around the middle of calf and thigh^44^. We estimated the isokinetic strength of the quadriceps muscle to obtain the muscle strength. A Kin Com MP isokinetic dynamometer (Chattanooga, TN) was used to assess the maximum voluntary concentric muscle force of the right knee extensor muscle with an angular velocity goal of 60 degrees/second^45^. The appendicular and lower limb lean masses were determined for the whole body by dual-energy X-ray absorptiometry (DXA; Hologic QDR-4500A scanner; Hologic, Waltham, MA, USA). DXA supplied validated total and regional measures of fat mass, bone mass, and lean mass in all age groups^46^. The participants received whole-body scans while lying in the supine position on the DXA table with their limbs close to their body. Body compositions were determined with the Hologic software version 11.2:3 (Hologic) for Windows^47^.

### Criteria of dynapenia and sarcopenia

To define dynapenia, the participants are divided into quartiles according to their sex-specific age, height-adjusted fat mass and leg strength residual values^48^. The isokinetic strength of the quadriceps muscle was used to determine the muscle strength. The patients in the lowest leg strength tertiles were defined as dynapenia.

For the definition of sarcopenia, the measure of DXA was performed to estimate the lean muscle mass in our study. The isokinetic strength of the quadriceps muscle was used to determine the muscle strength.

### Covariates

The covariates included those taken from the analysis of the self-report demographic information including age and race or ethnicity. The body mass index (BMI) was obtained based on the formula in which the weight of subject in kilograms is divided by the square of the height in meters (kg/m2). The laboratory data analyzed in the study included levels of serum aspartate aminotransferase(AST), total cholesterol, serum high-density lipoprotein (HDL), serum fasting glucose, serum calcium and serum creatinine. All measurements were collected with standard methods as delineated in a CDC reference, and all information is accessible from the NHANES database. For the comorbidities related to physical function, a trained interviewer confirmed the smoking status of the participants by asking the question “Do you smoke cigarettes now?”. The presences of congestive heart failure, coronary heart disease, angina/ angina pectoris, heart attack, stroke and arthritis were defined according to whether the participants had ever been diagnosed with or told that they had these conditions. All protocols followed the standardized methods based on the CDC reference methods. All experimental methods were performed in accordance with the relevant guidelines and regulations of CDC.

### Statistical Analysis

All statistical estimations were performed using by the Statistical Package for the Social Sciences, version18.0 (SPSS Inc., Chicago, IL, USA) for Windows. One-way analyses of variance and Pearson’s chi-square tests were performed to examine the differences between the groups in terms of demographic information, anthropometric parameters, laboratory data, and past histories. A two-sided *p*-value of ≤0.05 was regarded as the threshold for statistical significance. To prevent the influence of underlying comorbidities, we excluded all participants with abnormal serum phosphate levels and further divided the participants into quartiles based on their serum phosphate levels. When the participants in the lowest serum phosphate quartiles were regarded as the reference group, multivariate linear regression and logistic regression analyses were used. The following three-model approach was applied for covariate adjustment: Model 1 = unadjusted; Model 2 = age, gender, race, BMI, serum fasting glucose, serum cholesterol, AST, HDL, serum calcium and serum creatinine were included as covariates; and Model 3 = Model 2 + history of arthritis, congestive heart failure, coronary heart disease, stroke, and smoking were included as covariates.

## Electronic supplementary material

Supplementary Information
