# Supplementary material for: Exploring the Link between Serum Phosphate Levels and Low Muscle Strength, Dynapenia, and Sarcopenia
Source: Sci Rep. 2018 Feb 23;8:3573. doi: 10.1038/s41598-018-21784-1 (PMC5824959; doi:10.1038/s41598-018-21784-1)
Supplement: Supplementary file 1 — Supplementary Information [file 41598_2018_21784_MOESM1_ESM.doc]

**Exploring the Link between Serum Phosphate Levels and Low Muscle Strength, Dynapenia, and Sarcopenia**

Yuan-Yuei Chen, Tung-Wei Kao, Cheng-Wai Chou, Chen-Jung Wu, Hui-Fang Yang, Ching-Huang Lai, Li-Wei Wu, Wei-Liang Chen

Supplementary Table 1. Association between the serum phosphate and the anthropometric parameters.

| Anthropo-  metric Parameters | Model ^a^ 1  β^b^ (95% CI) | *P*  Value | Model ^a^ 2  β^b^ (95% CI) | *P*  Value | Model ^a^ 3  β^b^ (95% CI) | *P*  Value |
| --- | --- | --- | --- | --- | --- | --- |
| Muscle  Strength(nt) | -33.95  (-54.38, -13.52) | <0.001 | -31.17  (-51.63, -10.71) | 0.003 | -31.20  (-51.66, -10.75) | 0.003 |
| Appendicular lean mass. | -645.30  (-1205.03, -85.58) | 0.024 | 194.13  (-185.89, 574.15) | 0.317 | 201.95  (-178.60, 582.50) | 0.298 |
| TC(cm) | -2.34  (-3.20, -1.47) | <0.001 | -1.12  (-1.54, -0.70) | <0.001 | -1.09  (-1.51, -0.67) | <0.001 |
| CC(cm) | -1.27  (-1.80, -0.73) | <0.001 | -0.29  (-0.61, 0.21) | 0.068 | -0.27  (-0.59, 0.04) | 0.090 |

^a^ Adjusted covariates:

Model 1 = Unadjusted

Model 2 = Model 1 + age, sex, race/ethnicity, BMI, systolic blood pressure, serum fasting glucose, serum cholesterol, serum albumin, ALT, serum uric acid, C-reactive protein

Model 3 = Model 2 + history of congestive heart failure, coronary heart disease, angina/angina pectoris, heart attack, stroke, cancer/malignancy, smoking, moderate to vigorous recreational activity

^b^ β coefficients was interpreted as change of telomere length for each increase in different anthropometric parameters

Abbreviation:

TC, thigh circumference; CC, calf circumference

Supplementary Table 2. Characteristics of study participants of quartiles of serum phosphate

|  | Quartiles of serum phosphate | | | | | |
| --- | --- | --- | --- | --- | --- | --- |
| Characteristics of Study Participants | Q1 (0.55-1.03)  (n = 2030) | Q2 (1.03-1.16)  (n = 2018) | Q3 (1.16-1.26)  (n = 1869) | Q4 (1.26-2.39)  (n = 1900) | Total  (n=7817) | *P* Value |
| Continuous variables, mean (SD) | | | | | | |
| Age (years) | 52.61 (18.01) | 50.51 (18.79) | 48.65 (19.01) | 45.62 (18.81) | 49.42 (18.82) | <0.001 |
| Thigh circumference (cm) | 52.99 (7.08) | 53.12 (7.21) | 52.66 (7.01) | 52.81 (7.06) | 52.90 (7.09) | 0.212 |
| Calf circumference (cm) | 38.32 (4.33) | 38.36 (4.31) | 37.96 (4.25) | 38.01 (4.31) | 38.17 (4.33) | 0.004 |
| Appendicular lean mass. | 22477 (5870) | 21988 (6168) | 21335 (6082) | 21564 (6479) | 21865 (6158) | <0.001 |
| Muscle Strength (nt) | 369.06 (121.06) | 350.09 (124.92) | 338.14 (115.83) | 329.33 (124.62) | 348.87 (122.45) | <0.001 |
| BMI (kg/m^2^) | 28.81 (6.14) | 28.36 (6.20) | 28.07 (6.04) | 28.04 (6.21) | 28.33 (6.16) | <0.001 |
| AST (U/L) | 25.31 (19.42) | 24.24 (12.81) | 24.71 (24.12) | 24.70 (15.54) | 24.74 1(18.39) | 0.327 |
| Serum TC (mg/dL) | 199.23 (40.80) | 198.54 (38.64) | 201.86 (42.94) | 202.90 (42.49) | 200.57 (41.23) | 0.002 |
| Serum FG (mg/dL) | 102.24 (39.43) | 96.28 (30.52) | 96.18 (35.88) | 94.41 (34.26) | 97.35 (35.30) | <0.001 |
| Serum HDL (mg/dL) | 49.79 (15.02) | 51.60 (15.89) | 52.54 (15.56) | 52.71 (15.97) | 51.63 (15.65) | <0.001 |
| Calcium (mg/dL) | 9.31 (0.43) | 9.39 (0.39) | 9.43 (0.39) | 9.47 (0.41) | 9.40 (0.41) | <0.001 |
| Creatinine (mg/dL) | 0.81 (0.27) | 0.84 (0.39) | 0.80 (0.42) | 0.90 (0.76) | 0.84 (0.49) | <0.001 |
| Categorical variables, n (%) | | | | | | |
| Arthritis | 529 (26.1) | 509 (25.2) | 439 (23.5) | 415(21.8) | 1892 (24.2) | <0.001 |
| Congestive heart failure | 71 (3.5) | 54 (2.7) | 56 (3.0) | 48 (2.5) | 229 (2.9) | 0.363 |
| Coronary heart disease | 105 (5.2) | 83 (4.1) | 73 (3.9) | 67 (3.5) | 328 (4.2) | 0.104 |
| Angina/ angina pectoris | 82 (4.0) | 76 (3.8) | 67 (3.6) | 53 (2.8) | 278 (3.6) | 0.964 |
| Heart attack | 99 (4.9) | 83 (4.1) | 86 (4.6) | 67 (3.5) | 335 (4.3) | 0.082 |
| Stroke | 71 (3.5) | 59 (2.9) | 49 (2.6) | 58 (3.1) | 237 (3.0) | 0.367 |
| Smoking | 993 (48.9) | 973 (48.3) | 865 (46.3) | 961 (50.6) | 3792 (48.5) | 0.661 |
| Moderate to vigorous recreational activity | 1103 (54.3) | 1112 (55.1) | 1042 (55.8) | 1043 (54.9) | 4300 (55.0) | 0.637 |

BMI, body mass index; SBP, systolic blood pressure; Serum FG, serum fasting glucose; Serum TC, serum total cholesterol; AST, aspartate aminotransferase; Serum HDL, serum high density lipoprotein

^a^ Values were expressed as mean (standard deviation)

^b^ Values in the categorical variables were expressed as number (%)

| Variables | | Models | | | | | | | | | |
| --- | --- | --- | --- | --- | --- | --- | --- | --- | --- | --- | --- |
|  |  | Model 1 | | | Model 2 | | | Model 3 | | | |
| Anthropometric  parameters | Quartiles | β^b^ (95% CI) | *P* Value | *P* for Trend | β^b^ (95% CI) | *P* Value | *P* for Trend | β^b^ (95% CI) | *P* Value | *P* for Trend |  |
| Thigh Circumstance | Q2 v.s. Q1  Q3 v.s. Q1  Q4 v.s. Q1 | -0.27(-0.70, 0.15)  -0.83(-1.26, 0.39)  -1.05(-1.48, -0.61) | 0.208  <0.001  <0.001 |  | 0.09(-0.11, 0.29)  -0.12(-0.32, 0.09)  -0.38(-0.59, -0.17) | 0.363  0.257  <0.001 |  | 0.10(-0.10, 0.30)  -0.12(-0.32, 0.09)  -0.36(-0.57, -0.15) | 0.346  0.271  <0.001 |  |  |
| Calf Circumstance | Q2 v.s. Q1  Q3 v.s. Q1  Q4 v.s. Q1 | -0.09(-0.35, 0.18)  -0.46(-0.73, -0.19)  -0.54(-0.81, -0.27) | 0.510  <0.001  <0.001 |  | 0.18(0.03, 0.33)  -0.01(-0.16, 0.15)  -0.04(-0.19, 0.12) | 0.021  0.973  0.633 |  | 0.18(0.03, 0.33)  0.00(-0.15, 0.15)  -0.03(-0.18, 0.13) | 0.013  0.996  0.736 |  |  |
| Muscle Strength | Q2 v.s. Q1  Q3 v.s. Q1  Q4 v.s. Q1 | -8,69(-17.78, 0.40)  -10.59(-20.04, -1.13)  -14.14(-24.22, -4.07) | 0.061  0.028  0.006 |  | -7.72(-16.59, 1.14)  -9.44(-18.69, -0.20)  -13.01(-22.97, -3.06) | 0.088  0.045  0.010 |  | -7.14(-16.01, 1.73)  -0.90(-18.24, 0.24)  -12.88(-22.84, -2.92) | 0.114  0.056  0.011 |  |  |

Supplementary Table 3. Association between the Muscle strength and serum phosphate

^a^ Adjusted covariates:

Model 1 = Unadjusted

Model 2 = Model 1 + age, sex, race/ethnicity +BMI, serum fasting glucose, serum cholesterol, AST, serum HDL, serum calcium, serum creatinine

Model 3 = Model 2 + history of congestive heart failure, coronary heart disease, angina/angina pectoris, heart attack, stroke, smoking

^b^ β coefficients was interpreted as change of telomere length for each increase in different anthropometric parameters

Abbreviation: BMI, body mass index; SBP, systolic blood pressure; ALT, alanine aminotransferase

Supplementary Table 4. Association between the serum phosphate and the muscle strength in elderly participants

| Anthropo-  metric Parameters | Model ^a^ 1  β^b^ (95% CI) | *P*  Value | Model ^a^ 2  β^b^ (95% CI) | *P*  Value | Model ^a^ 3  β^b^ (95% CI) | *P*  Value |
| --- | --- | --- | --- | --- | --- | --- |
| <65 y/o | | | | | | |
| Muscle  Strength | -15.68  (-49.64, 18.28) | 0.365 | -23.48  (-57.24, 10.29) | 0.173 | -21.52  (-55.11, 12.06) | 0.209 |
| ≥65 y/o | | | | | | |
| Muscle  Strength | -39.43  (-69.79, -9.06) | 0.011 | -34.92  (-64.68, -5.16) | 0.021 | -35.17  (-65.01, -5.33) | 0.021 |

Model 1 = Unadjusted

Model 2 = Model 1 + age, sex, race/ethnicity +BMI, serum fasting glucose, serum cholesterol, AST, serum HDL, serum calcium, serum creatinine

Model 3 = Model 2 + history of congestive heart failure, coronary heart disease, angina/angina pectoris, heart attack, stroke, smoking

| Variables | | Models | | | | | |
| --- | --- | --- | --- | --- | --- | --- | --- |
|  |  | Model 1 | | Model 2 | | Model 3 | |
| Dynapenia | | | | | | | |
| Age group | Quartiles | OR  (95% CI) | *P* Value | OR  (95% CI) | *P* Value | OR  (95% CI) | *P* Value |
| 20-65 years old | Q2 v.s. Q1  Q3 v.s. Q1  Q4 v.s. Q1 | 1.37(0.99-1.89)  1.69(1.24-2.31)  1.60(1.15-2.24) | 0.06  <0.001  0.006 | 1.42(1.02-1.99)  1.74(1.26-2.40)  1.82(1.28-2.60) | 0.04  <0.001  <0.001 | 1.37(0.98-1.93)  1.67(1.20-2.32)  1.80(1.25-2.58) | 0.07  0.003  <0.001 |
| >65 years old | Q2 v.s. Q1  Q3 v.s. Q1  Q4 v.s. Q1 | 1.62(1.16-2.27)  1.86(1.31-2.62)  2.25(1.55-3.27) | 0.005  <0.001  <0.001 | 1.64(1.16-2.33)  1.76(1.23-2.51)  2.24(1.52-3.31) | 0.006  0.002  <0.001 | 1.57(1.10-2.23)  1.71(1.19-2.46)  2.13(1.43-3.16) | 0.013  0.004  <0.001 |
| Sarcopenia | | | | | | | |
| Age group | Quartiles | OR  (95% CI) | *P* Value | OR  (95% CI) | *P* Value | OR  (95% CI) | *P* Value |
| 20-65 years old | Q2 v.s. Q1  Q3 v.s. Q1  Q4 v.s. Q1 | 1.25(1.04-1.49)  1.31(1.09-1.57)  1.40(1.16-1.69) | 0.017  0.004  <0.001 | 1.15(0.92-1.44)  1.04(0.83-1.40)  1.18(0.93-1.50) | 0.210  0.728  0.167 | 1.15(0.92-1.43)  1.04(0.83-1.30)  1.18(0.93-1.50) | 0.225  0.747  0.169 |
| >65 years old | Q2 v.s. Q1  Q3 v.s. Q1  Q4 v.s. Q1 | 1.21(1.00-1.47)  1.34(1.11-1.63)  1.48(1.23-1.79) | 0.047  0.002  <0.001 | 1.09(0.86-1.37)  1.01(0.80-1.28)  1.23(0.97-1.56) | 0.475  0.934  0.089 | 1.09(0.86-1.38)  1.00(0.79-1.27)  1.24(0.98-1.57) | 0.468  0.978  0.080 |

Supplementary Table 5. Association between the quartiles of serum phosphate and the presence of dynapenia and sarcopenia

Model 1 = Unadjusted

Model 2 = Model 1 + age, sex, race/ethnicity +BMI, fasting glucose, serum cholesterol, AST, serum HDL, serum calcium, serum creatinine

Model 3 = Model 2 + history of congestive heart failure, coronary heart disease, angina/angina pectoris, heart attack, stroke, smoking

Supplementary Table 6. Association between the serum phosphate and the anthropometric parameters.

| Anthropo-  metric Parameters | Model ^a^ 1  β^b^ (95% CI) | *P*  Value | Model ^a^ 2  β^b^ (95% CI) | *P*  Value | Model ^a^ 3  β^b^ (95% CI) | *P*  Value |
| --- | --- | --- | --- | --- | --- | --- |
| Muscle  strength | -24.02  (-47.15, -0.89) | 0.042 | -24.28  (-47.10, -1.47) | 0.037 | -24.23  (-47.04, -1.42) | 0.037 |
| Appendicular lean mass. | -868.54  (-1529.34, -207.74) | 0.010 | 217.00  (-220.24, 654.25) | 0.331 | 222.70  (-214.84, 660.24) | 0.318 |
| Lower limbs lean mass | -791.44  (-1285.30, -297.58) | 0.002 | 74.25  (-251.48, 399.99) | 0.665 | 80.99  (-244.91, 406.91 | 0.626 |
| TC | -2.40  (-3.43, -1.37) | <0.001 | -0.79  (-1.28, -0.31) | <0.001 | -0.77  (-1.26, -0.28) | 0.002 |
| CC | -1.43  (-2.06, -0.79) | <0.001 | -0.25  (-0.62, -0.12) | 0.178 | -0.23  (-0.60, 0.14 | 0.221 |

Model 1 = Unadjusted

Model 2 = Model 1 + age, sex, race/ethnicity +BMI, serum fasting glucose, cholesterol, AST, HDL, calcium, creatinine

Model 3 = Model 2 + history of arthritis, congestive heart failure, coronary heart disease, angina/angina pectoris, heart attack, stroke, smoking

Abbreviation:

TC, thigh circumference; CC, calf circumference

Supplementary Table 7. Characteristics of study participants of quartiles of serum phosphate in male participants

|  | Quartiles of serum phosphate | | | | | |
| --- | --- | --- | --- | --- | --- | --- |
| **Male** | | | | | | |
| Characteristics of Study Participants | Q1 (0.81-1.03)  (n = 1077) | Q2 (1.03-1.13)  (n = 1013) | Q3 (1.13-1.23)  (n = 812) | Q4 (1.23-1.49)  (n = 669) | Total  (n=3571) | *P* Value |
| Continuous variables, mean (SD) | | | | | | |
| Age (years) | 54.56(17.93) | 51.51(18.60) | 48.99(18.38) | 45.44(17.80) | 50.72(18.48) | <0.001 |
| Thigh circumference (cm) | 52.35(6.01) | 52.78(6.21) | 52.54(6.23) | 53.42(6.62) | 52.72(6.24) | 0.006 |
| Calf circumference (cm) | 38.33(3.89) | 38.56(3.93) | 38.29(3.99) | 38.76(4.10) | 38.47(3.96) | 0.075 |
| Appendicular lean mass. | 25451(4802) | 25932(4994) | 25659(5117) | 26442(5387) | 25821(5053) | <0.001 |
| Muscle Strength (nt) | 411.24(114.59) | 411.95(120.90) | 405.65(118.64) | 417.33(132.44) | 411.11(119.83) | 0.783 |
| BMI (kg/m^2^) | 28.08(5.15) | 27.86(5.39) | 27.55(5.31) | 27.85(5.60) | 27.85(5.34) | 0.225 |
| AST (U/L) | 29.48(22.20) | 28.76(21.32) | 30.88(44.20) | 32.19(27.37) | 30.10(29.38) | 0.087 |
| Serum TC (mg/dL) | 198.36(40.08) | 196.14(39.11) | 198.71(41.72) | 196.76(40.81) | 197.51(40.32) | 0.458 |
| Serum FG (mg/dL) | 102.69(34.58) | 99.94(35.53) | 98.84(36.22) | 97.58(37.49) | 100.08(35.81) | 0.019 |
| Serum HDL (mg/dL) | 49.79 (15.02) | 51.60 (15.89) | 52.54 (15.56) | 52.71 (15.97) | 51.63 (15.65) | <0.001 |
| Calcium (mg/dL) | 9.36(0.40) | 9.46(0.38) | 9.50(0.37) | 9.52(0.38) | 9.45(0.39) | <0.001 |
| Creatinine (mg/dL) | 0.91(0.24) | 0.97(0.47) | 0.95(0.29) | 1.03(0.60) | 0..96(0.41) | <0.001 |
| Categorical variables, n (%) | | | | | | |
| Arthritis | 263(24.4) | 218(21.5) | 158(19.5) | 125(18.7) | 764(21.4) | <0.001 |
| Congestive heart failure | 42(3.9) | 28(2.8) | 28(3.4) | 16(2.4) | 114(3.2) | 0.571 |
| Coronary heart disease | 76(7.1) | 58(5.7) | 43(5.3) | 28(4.2) | 205(5.7) | 0.018 |
| Angina/ angina pectoris | 49(4.5) | 44(4.3) | 31(3.8) | 21(3.1) | 145(4.1) | 0.799 |
| Heart attack | 71(6.6) | 58(5.7) | 52(6.4) | 37(5.5) | 218(6.1) | 0.841 |
| Stroke | 40(3.7) | 33(3.3) | 21(2.6) | 25(3.7) | 119(3.3) | 0.781 |
| Smoking | 630(58.6) | 604(59.7) | 482(59.4) | 423(63.2) | 2139(59.9) | 0.168 |
| Moderate to vigorous recreational activity | 567(52.6) | 556(54.9) | 443(54.6) | 376(56.2) | 1942(54.4) | 0.170 |

BMI, body mass index; SBP, systolic blood pressure; Serum FG, serum fasting glucose; Serum TC, serum total cholesterol; AST, aspartate aminotransferase; Serum HDL, serum high density lipoprotein

^a^ Values were expressed as mean (standard deviation)

^b^ Values in the categorical variables were expressed as number (%)

Supplementary Table 8. Characteristics of study participants of quartiles of serum phosphate in female participants

|  | Quartiles of serum phosphate | | | | | |
| --- | --- | --- | --- | --- | --- | --- |
| **Female** | | | | | | |
| Characteristics of Study Participants | Q1 (0.81-1.06)  (n = 803) | Q2 (1.03-1.16)  (n = 1055) | Q3 (1.13-1.29)  (n = 1057) | Q4 (1.29-1.49)  (n = 985) | Total  (n=3850) | *P* Value |
| Continuous variables, mean (SD) | | | | | | |
| Age (years) | 49.94(18.14) | 49.50(18.93) | 48.38(19.49) | 46.84(19.67) | 48.60(19.15) | 0.002 |
| Thigh circumference (cm) | 53.84(8.20) | 53.45(8.09) | 52.74(7.56) | 52.27(7.32) | 53.04(7.80) | <0.001 |
| Calf circumference (cm) | 38.34(4.80) | 38.16(4.64) | 37.71(4.43) | 37.42(4.48) | 37.88(4.59) | <0.001 |
| Appendicular lean mass. | 18110(4325) | 17699(4123) | 17631(4064) | 17362(3784) | 17689(4079) | 0.004 |
| Muscle Strength (nt) | 287.27(83.30) | 276.66(82.84) | 283.70(79.18) | 277.45(81.94) | 281.17(81.72) | 0.339 |
| BMI (kg/m^2^) | 29.75(7.08) | 28.86(6.88) | 28.47(6.51) | 28.07(6.60) | 28.74(6.78) | <0.001 |
| AST (U/L) | 21.05(16.27) | 20.65(13.18) | 21.52(26.11) | 21.04(12.22) | 21.07(18.05) | 0.748 |
| Serum TC (mg/dL) | 202.07(41.78) | 200.96(38.03) | 204.28(43.71) | 206.06(43.63) | 203.41(41.38) | 0.031 |
| Serum FG (mg/dL) | 100.53(45.31) | 92.60(23.93) | 94.14(35.51) | 93.07(32.82) | 94.80(34.75) | <0.001 |
| Serum HDL (mg/dL) | 49.79 (15.02) | 51.60 (15.89) | 52.54 (15.56) | 52.71 (15.97) | 51.63 (15.65) | <0.001 |
| Calcium (mg/dL) | 9.26(0.46) | 9.32(0.40) | 9.37(0.39) | 9.42(0.40) | 9.35(0.42) | <0.001 |
| Creatinine (mg/dL) | 0.66(0.23) | 0.70(0.21) | 0.70(0.47) | 0.73(0.31) | 0.70(0.33) | <0.001 |
| Categorical variables, n (%) | | | | | | |
| Arthritis | 227(28.3) | 291(29.0) | 281(26.6) | 244(24.8) | 1043(27.1) | 0.020 |
| Congestive heart failure | 22(2.7) | 26(2.6) | 28(2.6) | 18(1.8) | 94(2.4) | 0.180 |
| Coronary heart disease | 22(2.7) | 25(2.5) | 30(2.8) | 22(2.2) | 99(2.6) | 0.790 |
| Angina/ angina pectoris | 25(3.1) | 32(3.2) | 36(3.4) | 18(1.8) | 111(2.9) | 0.850 |
| Heart attack | 19(2.4) | 25(2.5) | 34(3.2) | 16(1.6) | 94(2.4) | 0.299 |
| Stroke | 24(3.0) | 26(2.6) | 28(2.6) | 30(3.0) | 108(2.8) | 0.465 |
| Smoking | 294(36.6) | 369(36.8) | 383(36.3) | 405(41.1) | 1451(37.7) | 0.111 |
| Moderate to vigorous recreational activity | 455(56.7) | 556(55.3) | 599(56.7) | 524(53.2) | 2134(55.4) | 0.223 |

BMI, body mass index; SBP, systolic blood pressure; Serum FG, serum fasting glucose; Serum TC, serum total cholesterol; AST, aspartate aminotransferase; Serum HDL, serum high density lipoprotein

^a^ Values were expressed as mean (standard deviation)

^b^ Values in the categorical variables were expressed as number (%)

Supplementary Table 9. Association between the anthropometric parameters and quartiles of serum phosphate

| Variables | | Models | | | | | | | | | | |
| --- | --- | --- | --- | --- | --- | --- | --- | --- | --- | --- | --- | --- |
|  |  | Model 1 | | | Model 2 | | | | Model 3 | | | |
| Anthropometric  parameters | Quartiles | β^b^ (95% CI) | *P* Value | *P* for Trend | β^b^ (95% CI) | *P* Value | *P* for Trend | β^b^ (95% CI) | | *P* Value | *P* for Trend |  |
| Thigh Circumstance | Q2 v.s. Q1  Q3 v.s. Q1  Q4 v.s. Q1 | -0.26(-0.69, 0.18)  -0.81(-1.26, -0.37)  -1.00(-1.46, -0.54) | 0.247  <0.001  <0.001 | <0.001 | 0.07(-0.13, 0.27)  -0.14(-0.35, 0.06)  -0.32(-0.54, -0.10) | 0.491  0.173  0.004 | <0.001 | 0.07(-0.13, 0.28)  -0.14(-0.35, 0.07)  -0.31(-0.52, -0.09) | | 0.472  0.182  0.006 | <0.001 |  |
| Calf Circumstance | Q2 v.s. Q1  Q3 v.s. Q1  Q4 v.s. Q1 | -0.09(-0.36, 0.18)  -0.47(-0.74, -0.19)  -0.55(-0.83, -0.26) | 0.504  <0.001  <0.001 | <0.001 | -0.15(-0.01, 0.30)  -0.03(-0.19, 0.13)  -0.04(-0.21, 0.12) | 0.054  0.703  0.602 | 0.228 | 0.154(0.01, 0.31)  -0.03(-0.18, 0.13)  -0.03(-0.20, 0.13) | | 0.047  0.730  0.699 | 0.278 |  |
| Muscle Strength | Q2 v.s. Q1  Q3 v.s. Q1  Q4 v.s. Q1 | -7.66(-16.89, 1.57)  -9.62(-19.20, -0.04)  -10.47(-20.92, -0.01) | 0.104  0.049  0.050 | 0.033 | -7.08(-16.08, 1.92)  -8.90(-18.28, 0.47)  -10.44(-20.71, -0.16) | 0.123  0.063  0.047 | 0.034 | -6.48(-15.48, 2.52)  -8.47(-17.84, 0.89)  -10.39(-20.66, -0.12) | | 0.158  0.076  0.047 | 0.035 |  |

Model 1 = Unadjusted

Model 2 = Model 1 + age, sex, race/ethnicity +BMI, serum fasting glucose, cholesterol, AST, HDL, calcium, creatinine

Model 3 = Model 2 + history of arthritis, congestive heart failure, coronary heart disease, angina/angina pectoris, heart attack, stroke, smoking

Supplementary Table 10. Association between the quartiles of serum phosphate and the muscle strength categorized by gender

|  | Quartiles | Model ^a^ 1  β^b^ (95% CI) | *P*  Value | Model ^a^ 2  β^b^ (95% CI) | *P*  Value | Model ^a^ 3  β^b^ (95% CI) | *P*  Value |
| --- | --- | --- | --- | --- | --- | --- | --- |
| Male | | | | | | | |
| Muscle  Strength | Q2 vs Q1  Q3 vs Q1  Q4 vs Q1 | 0.72(-15.66, 17.09)  -5.60(-23.53, 12.35)  6.09(-14.56, 26.74) | 0.932  0.541  0.563 | -6.45(-20.10, 7.20)  -16.54(-31.58, -1.51)  -17.24(-34.65, 0.16) | 0.354  0.031  0.052 | -6.39(-20.12, 7.33)  -16.86(-31.97, -1.74)  -17.53(-35.02, -0.05) | 0.361  0.029  0.049 |
| Female | | | | | | | |
| Muscle  Strength | Q2 vs Q1  Q3 vs Q1  Q4 vs Q1 | -10.61(-23.95, 2.73)  -3.57(-16.69, 9.54)  -9.83(-23.49, 3.84) | 0.119  0.593  0.159 | -13.47(-24.97, -1.96)  -8.59(-19.92, 2.74)  -13.80(-25.66, -1.94) | 0.022  0.137  0.023 | -13.14(-24.63, -1.64)  -8.72(-20.05, 2.61)  -14.41(-26.27, -2.56) | 0.025  0.131  0.017 |

Model 1 = Unadjusted

Model 2 = Model 1 + age, sex, race/ethnicity +BMI, serum fasting glucose, cholesterol, AST, HDL, calcium, creatinine

Model 3 = Model 2 + history of arthritis, congestive heart failure, coronary heart disease, angina/angina pectoris, heart attack, stroke, smoking

| Variables | | Models | | | | | |
| --- | --- | --- | --- | --- | --- | --- | --- |
|  |  | Model 1 | | Model 2 | | Model 3 | |
| Dynapenia | | | | | | | |
| Sex | Quartiles | OR  (95% CI) | *P* Value | OR  (95% CI) | *P* Value | OR  (95% CI) | *P* Value |
| Male | Q2 v.s. Q1  Q3 v.s. Q1  Q4 v.s. Q1 | 1.42(0.99, 2.05)  1.34(0.88, 2.02)  1.43(0.91, 2.25) | 0.059  0.169  0.121 | 1.76(1.15, 2.70)  1.71(1.05, 2.76)  2.15(1.25, 3.68) | 0.010  0.030  0.005 | 1.81(1.17, 2.78)  1.73(1.06, 2.82)  2.20(1.27, 3.81) | 0.007  0.027  0.005 |
| Female | Q2 v.s. Q1  Q3 v.s. Q1  Q4 v.s. Q1 | 1.19(0.73, 1.93)  1.13(0.71, 1.81)  1.62(0.96, 2.72) | 0.496  0.600  0.070 | 0.27(0.73, 2.34)  0.24(0.73, 2.23)  0.72(1.10, 3.82) | 0.366  0.391  0.022 | 0.25(0.71, 2.30)  0.25(0.73, 2.25)  0.76(1.14, 3.98) | 0.416 0.388  0.018 |
| Sarcopenia | | | | | | | |
| Sex | Quartiles | OR  (95% CI) | *P* Value | OR  (95% CI) | *P* Value | OR  (95% CI) | *P* Value |
| Male | Q2 v.s. Q1  Q3 v.s. Q1  Q4 v.s. Q1 | 0.94(0.62, 1.42)  1.12(0.73, 1.71)  0.95(0.60, 1.52) | 0.759  0.601  0.844 | 1.06(0.69, 1.62)  1.37(0.88, 2.12)  1.47(0.90, 2.39) | 0.791  0.159  0.124 | 1.07(0.70, 1.64)  1.39(0.90, 2.16)  1.39(0.84, 2.27) | 0.761  0.143  0.198 |
| Female | Q2 v.s. Q1  Q3 v.s. Q1  Q4 v.s. Q1 | 1.17(0.95, 1.44)  1.14(0.92, 1.40)  1.29(1.04, 1.60) | 0.139  0.227  0.018 | 1.21(0.96, 1.51)  1.14(0.92, 1.43)  1.43(1.13, 1.80) | 0.101  0.249  0.003 | 1.21(0.96, 1.51)  1.14(0.91, 1.43)  1.43(1.13, 1.81) | 0.101  0.250  0.003 |

Supplementary Table 11. Logistic regression for the association of quartiles of serum phosphate and the presence of low muscle strength

Model 1 = Unadjusted

Model 2 = Model 1 + age, sex, race/ethnicity +BMI, serum fasting glucose, cholesterol, AST, HDL, calcium, creatinine

Model 3 = Model 2 + history of arthritis, congestive heart failure, coronary heart disease, angina/angina pectoris, heart attack, stroke, smoking

Supplementary Table 12. Association between the serum phosphate and the muscle strength in the NHANES 2000-2001

| Anthropo-  metric Parameters | Model ^a^ 1  β^b^ (95% CI) | *P*  Value | Model ^a^ 2  β^b^ (95% CI) | *P*  Value | Model ^a^ 3  β^b^ (95% CI) | *P*  Value |
| --- | --- | --- | --- | --- | --- | --- |
| Muscle  strength | -99.61  (-138.18, -61.05) | <0.001 | -43.65  (-72.43, -14.86) | 0.003 | -43.81  (-72.74, -14.89) | 0.003 |

Model 1 = Unadjusted

Model 2 = Model 1 + age, sex, race/ethnicity +BMI, Vitamin D, serum fasting glucose, cholesterol, AST, HDL, calcium, creatinine

Model 3 = Model 2 + history of arthritis, congestive heart failure, coronary heart disease, angina/angina pectoris, heart attack, stroke, smoking

Abbreviation:

TC, thigh circumference; CC, calf circumference

Supplementary Table 13. Association between the quartiles of serum phosphate and the muscle strength in the NHANES 2000-2001

|  | Quartiles | Model ^a^ 1  β^b^ (95% CI) | *P*  Value | Model ^a^ 2  β^b^ (95% CI) | *P*  Value | Model ^a^ 3  β^b^ (95% CI) | *P*  Value |
| --- | --- | --- | --- | --- | --- | --- | --- |
| Male | | | | | | | |
| Muscle  Strength | Q2 vs Q1  Q3 vs Q1  Q4 vs Q1 | -26.24(-45.01, -7.48)  -32.39(-51.99, -12.78)  -47.82(-66.53, -29.11) | 0.006  <0.001  <0.001 | -13.31(-26.73, 0.11)  -12.16(-26.32, 1.99)  -19.78(-33.56, -5.99) | 0.052  0.092  0.005 | -13.33(-26.80, 0.14)  -12.70(-26.92, 1.52)  -19.81(-33.66, -5.97) | 0.052  0.080  0.005 |

Model 1 = Unadjusted

Model 2 = Model 1 + age, sex, race/ethnicity +BMI, Vitamin D, serum fasting glucose, cholesterol, AST, HDL, calcium, creatinine

Model 3 = Model 2 + history of arthritis, congestive heart failure, coronary heart disease, angina/angina pectoris, heart attack, stroke, smoking

Supplementary Table 14. Association between the serum phosphate and the muscle strength categorized by different level of C-reactive protein (CRP) (cut-off points=0.49)

|  | | Model ^a^ 1  β^b^ (95% CI) | *P*  Value | Model ^a^ 2  β^b^ (95% CI) | *P*  Value | Model ^a^ 3  β^b^ (95% CI) | *P*  Value |
| --- | --- | --- | --- | --- | --- | --- | --- |
| Muscle  Strength | Low  CRP | -75.03  (-113.36, -36.70) | <0.001 | -16.78  (-44.83, 11.28) | 0.241 | -16.64  (-44.75, 11.47) | 0.246 |
|  | High  CRP | -99.46  (-149.64, -49.27) | <0.001 | -65.49  (-106.07, -24.91) | 0.002 | -67.67  (-108.28, -27.06) | <0.001 |

Model 1 = Unadjusted

Model 2 = Model 1 + age, sex, race/ethnicity +BMI, serum fasting glucose, serum cholesterol, AST, serum HDL, serum calcium, serum creatinine

Model 3 = Model 2 + history of congestive heart failure, coronary heart disease, angina/angina pectoris, heart attack, stroke, smoking
